# A Wi-Fi–Based Mask-Type Laryngoscope for Telediagnosis During the COVID-19 Pandemic: Instrument Validation Study

**DOI:** 10.2196/31224

**Published:** 2021-10-18

**Authors:** Youngjin Moon, Jaeho Hyun, Jeongmin Oh, Kwanhee Lee, Yoon Se Lee, Jun Ki Kim

**Affiliations:** 1 Biomedical Engineering Research Center Asan Institute for Life Sciences Asan Medical Center Seoul Republic of Korea; 2 Department of Convergence Medicine University of Ulsan College of Medicine Seoul Republic of Korea; 3 Department of Biomedical Engineering University of Ulsan College of Medicine Seoul Republic of Korea; 4 Department of Otorhinolaryngology - Head and Neck Surgery Asan Medical Center Seoul Republic of Korea

**Keywords:** smartphone-based endoscope, mobile health, telediagnosis, continuum segment, articulable endoscope, COVID-19, point-of-care diagnostics, validation, medical device, endoscope, sensor, innovation, video, transmission

## Abstract

**Background:**

Owing to the COVID-19 pandemic, social distancing has become mandatory. Wireless endoscopy in contactless examinations promises to protect health care workers and reduce viral spread.

**Objective:**

This study aimed to introduce a contactless endoscopic diagnosis system using a wireless endoscope resembling a mask.

**Methods:**

The Wi-Fi–based contactless mask endoscopy system comprises a disposable endoscope and a controller. First, the effective force applied by the tip during insertion was evaluated in a simple transoral model consisting of a force sensor on a simulated oropharynx wall. Second, the delay in video streaming was evaluated by comparing the frame rate and delays between a movement and its image over direct and Wi-Fi connections. Third, the system was applied to a detailed laryngopharyngeal tract phantom.

**Results:**

The smartphone-controlled wireless endoscopy system was successfully evaluated. The mean, maximum, and minimum collision forces against the wall of the transoral model were 296 mN (30 gf), 363 mN (37 gf), and 235 mN (24 gf), respectively. The delay resulting from the wireless connection was 0.72 seconds. Using the phantom, an inexperienced user took around 1 minute to orient the endoscope to a desired area via the app.

**Conclusions:**

Device articulation does not pose a significant risk of laryngopharyngeal wall penetration, and latency does not significantly impede its use. Contactless wireless video streaming was successful within the access point range regardless of the presence of walls. The mask endoscope can be controlled and articulated wirelessly, minimizing contact between patients and device operators. By minimizing contact, the device can protect health care workers from infectious viruses like the coronavirus.

## Introduction

With the recent outbreak of COVID-19, the importance of quarantine and contact reduction has increased. Social distancing has become mandatory to lessen the impact of the outbreak and prevent the further spread of the virus, because increased contact with infected individuals drastically increases the possibility of infection. Those who work in the medical field are at particularly high risk of exposure. According to Nguyen et al, front-line health care workers are 4 to 10 times more likely to get the disease in the United States and the United Kingdom [1].

To protect health care workers and prevent further spread of the coronavirus, contactless examination has emerged. For contactless examination and treatment, governments worldwide have started implementing various contactless infrastructure and devices. For example, South Korean health care service workers use walk-through screening centers for examination [2]. Separated by a box, respiratory splash is completely prevented with a negative pressure applied inside the booth. Experts speculate that even after the pandemic ends, with COVID-19 being eradicated or neutralized like the common cold, there will always be a possibility of another pandemic [3]. Therefore, requirements for contactless devices will persistently rise after 2021 for all medical procedures that include the risk of possible viral infection, and endoscopy will not be an exception.

The endoscope is widely accepted as an examination device for a wide range of diseases. Starting as a rigid bronchoscope, over 100 years of development have enabled endoscopes to become flexible, therefore enhancing both patient experience and accessibility to internal organs [4,5]. However, in a pandemic, even a simple endoscopy can be a serious threat to patients. According to Kovaleva et al, several microorganisms can be transferred from one patient to another during endoscopy [6]. Most infections are via direct contact with incompletely disinfected devices, but viruses and other microorganisms can be transferred by not only an infected endoscope but also aerosols generated during the procedure [7]. Especially for highly infectious diseases like COVID-19, the entire facility involved in endoscopy can be at risk. To minimize contact with patients who undergo endoscopy, several prevention methods have been suggested, from minimizing the practice of endoscopy itself [8] to completely covering patients with protective sheets. However, since endoscopy has to be performed right beside the patient, it does not afford the same degree of safety to health care workers.

Wireless endoscopy has been around for a while, but has never been practically used. Most wireless endoscopes are capsule endoscopes, whose purpose is to specifically record the gastrointestinal tract, usually with control of the magnetic force applied from the outer belt patients are asked to wear [9]. There are several reasons wireless endoscopy is not favored over traditional, wired endoscopy, such as battery depletion and a low data transfer rate. If the endoscope is flexible, flexibility is usually manifested by tension applied to the string attached to a motor, transforming the position of individual segments of the continuum robot. The endoscope can be controlled directly using the controller attached to the motor. There is no reason to split the flexible endoscope into 2 parts and, therefore, discard the wired endoscope over the wireless endoscope, unless the desired area is too deep for the cable to reach. However, in dire situations when a contactless procedure should be the new norm, a wireless endoscope with articulation can be a huge advantage over a wired endoscope.

In this study, we introduced a novel, contactless endoscopic diagnosis system resembling a mask, enabling users to put it on easily themselves. The newly designed continuum part comprises 2 articulating units rolled into the device and sliding out when needed. While the distal tip unit freely articulates in the direction the user desires, the proximal tip holds down the user’s tongue, providing a better endoscopy experience. The endoscope unit is controlled by a mobile app, over a Wi-Fi connection. Once the patient wears the device, the system enables the operator to perform endoscopy without direct contact with the patient.

## Methods

### Development of a Wi-Fi–Based Contactless Mask Endoscope

**Figure 1:**
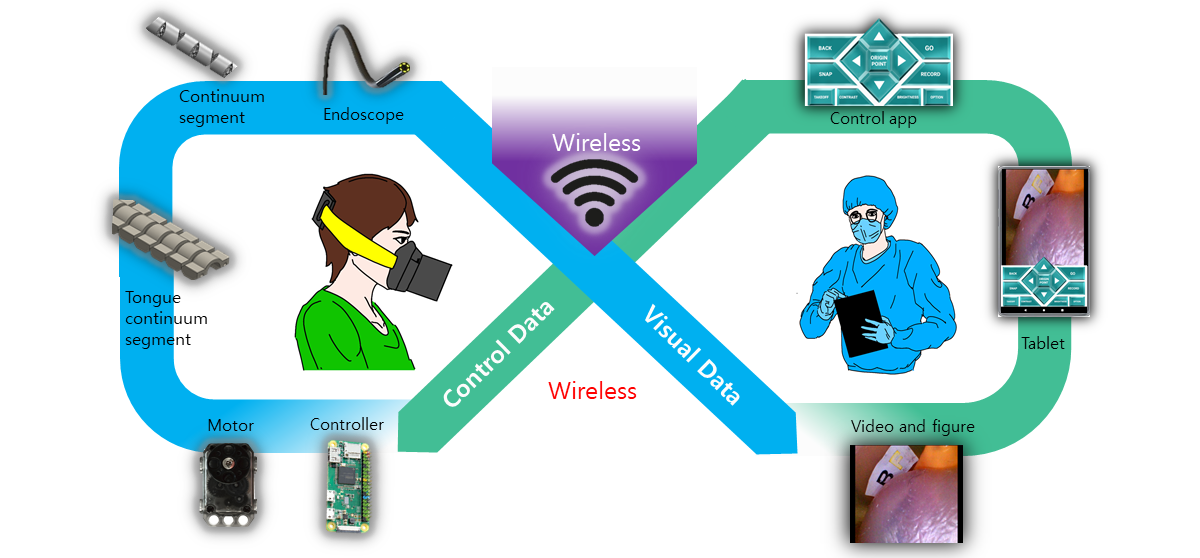
Overall scheme of the Wi-Fi–based contactless mask endoscopic system.

**Figure 2:**
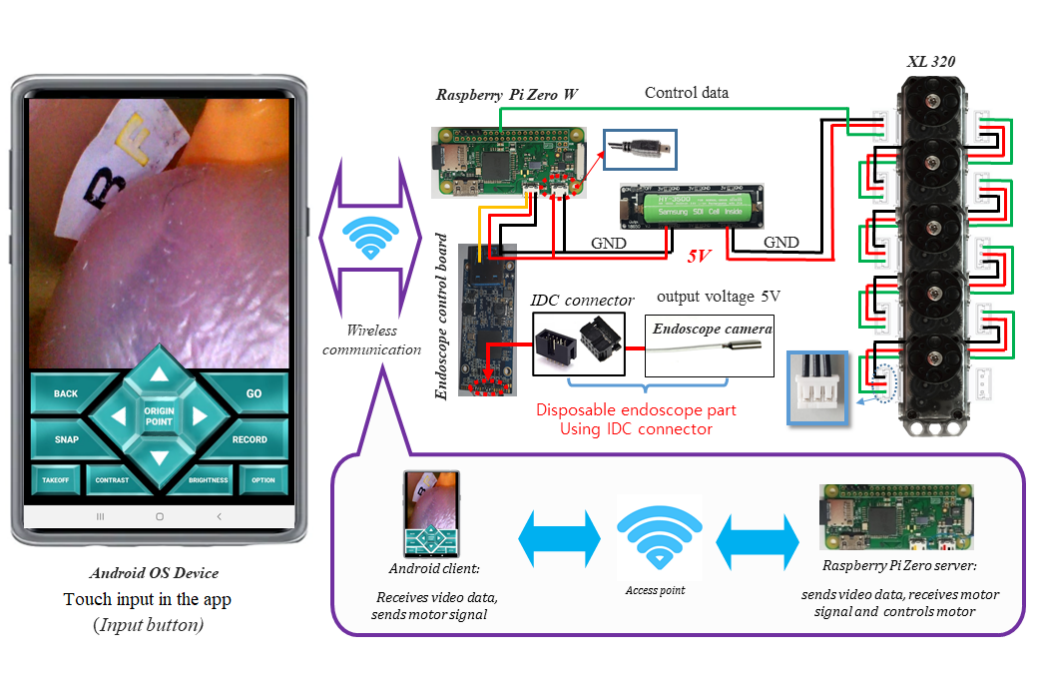
Electric wiring and key function of the Wi-Fi–based contactless mask endoscopic system. GND: ground; IDC: insulation-displacement contact.

We built a Wi-Fi–based contactless mask endoscopic system. Figure 1 depicts the overall scheme of how a Wi-Fi–based contactless mask endoscope works in the field. The device comprises 2 parts, a disposable endoscope and a controller. The disposable endoscope captures and transmits digital visual data to the controller. The controller comprises a continuum segment, motors, and a microprocessor. The controller and the operator’s Android mobile device are connected over a wireless network. The controller is responsible for articulation of the disposable endoscope and transmits visual data obtained from the endoscope to the connected mobile device. On the operator’s side, a specific app for the endoscope helps the operator interface with the device using a wireless connection. The app receives real-time visual data from the device, while it sends control data to the device. The communication was based on the connection between electrical components. Figure 2 describes the connection and general flow of data and electricity in the Wi-Fi–based contactless mask endoscope.

Being a portable device, the contactless mask endoscope draws electricity from a rechargeable lithium battery. Raspberry Pi Zero W, a microprocessor containing a Wi-Fi module and memory sufficient enough to serve as a simple Wi-Fi server, is the center of the device. The device is connected to 5 motors, which also draw electricity from the battery. The endoscope module is a separate pluggable module that can be unplugged for maintenance and cleaning. Connected to the endoscope control board, Raspberry Pi Zero W receives real-time video signals from the endoscope module. To communicate with each other, Raspberry Pi Zero W and the operator’s Android tablet are connected within the same network. The touch-based input of the app is converted into a directional signal in the app and sent to Raspberry Pi Zero W to manipulate the continuum segment.

**Figure 3:**
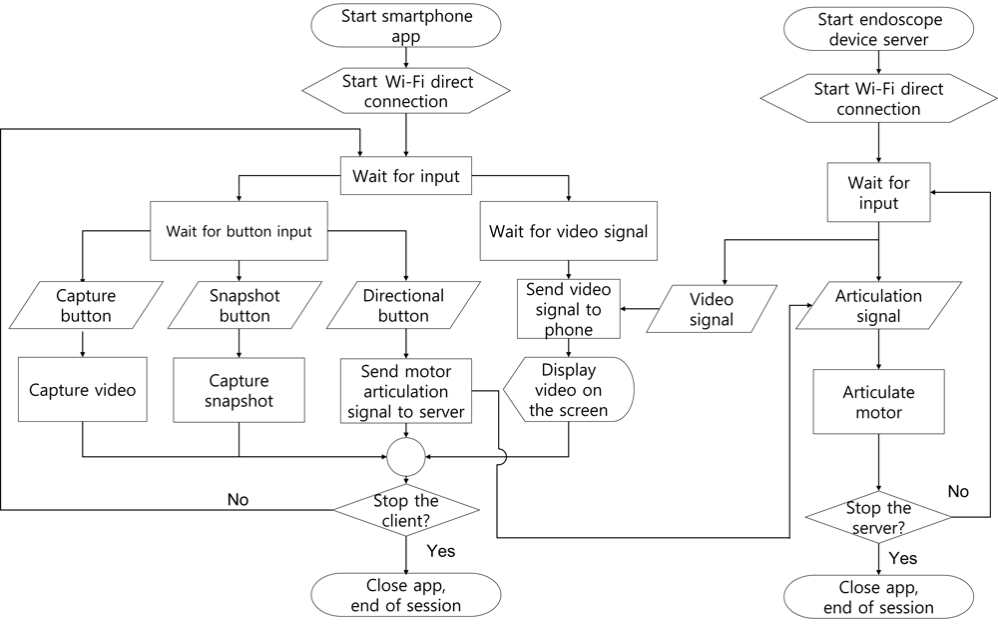
Flowchart of the software part of the Wi-Fi–based contactless mask endoscopic system in action.

The detailed Wi-Fi communication flow is described in Figure 3. Both the smartphone app and the device are booted, and they are manually connected to the same network. When the Wi-Fi connection is secured, the server is automatically started in the endoscope, while the app waits for the server connection. When the server successfully starts, it immediately starts to stream video information, while listening for directional button input from the client app. The client app starts to receive the video stream and readies to send a motor signal to the endoscope server. At this point, the user can easily send a direction-steering signal to the server by pressing the directional button on the app user interface. The app saves a snapshot and the video stream, without affecting the video stream. If the Wi-Fi connection is severed, the app and server revert to the waiting stage. If the user stops the session, the app is safe to turn off.

**Figure 4:**
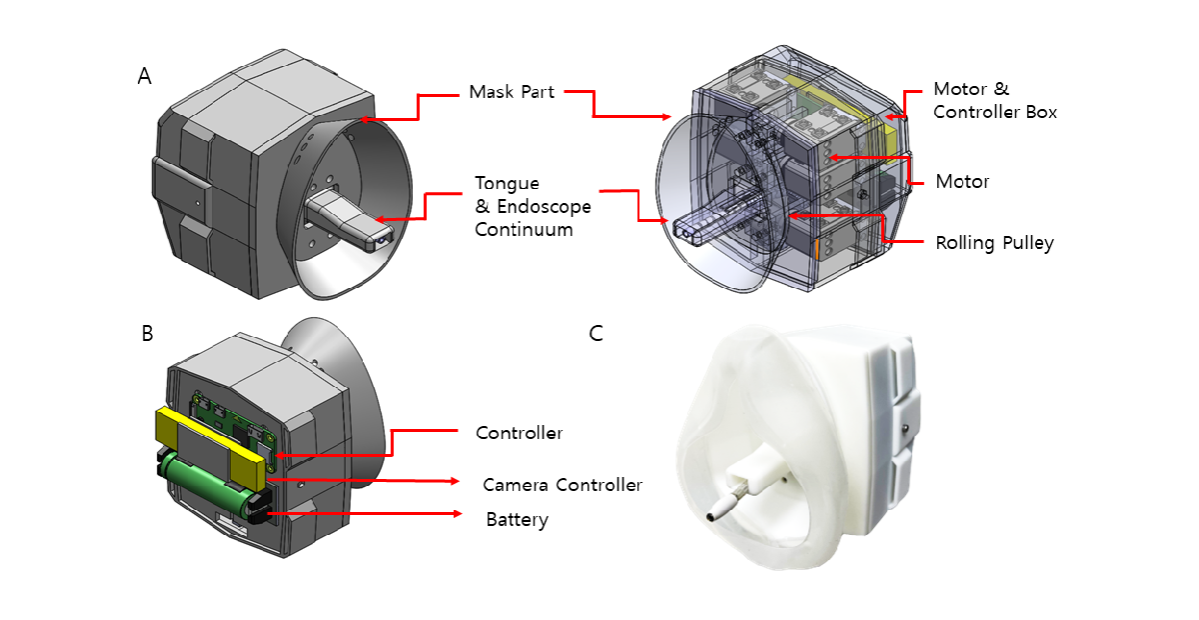
Assembled hardware parts in 3D space. (A) Rendered 3D structure of the outside shell and internal articulating parts of the hardware. (B) Position of the electronic parts of the hardware, rendered in 3D space. (C) Printed and assembled final product in real space.

The mechanical parts of the mask endoscope are roughly divided into a mask, a motor and controller box, and a continuum. The mask is where crucial systemic components are present. The lid of the mask can be removed to place a rechargeable battery and perform maintenance of the Raspberry server. Figure 4 shows the internal and external structure of the 3D mask endoscope in 3D space. The arrangement of the motor and controller box, pulley, and controlling parts are shown on the right of Figure 4A. The motors are separated from the controller by a partition. As shown in Figure 4B, the controllers and the battery are in the outermost side of the motor and controller box, easily accessible by removing the lid. Figure 4C shows a finished prototype in 3D space. In addition to the mask, a soft support is added to improve patient experience by enhancing texture.

**Figure 5:**
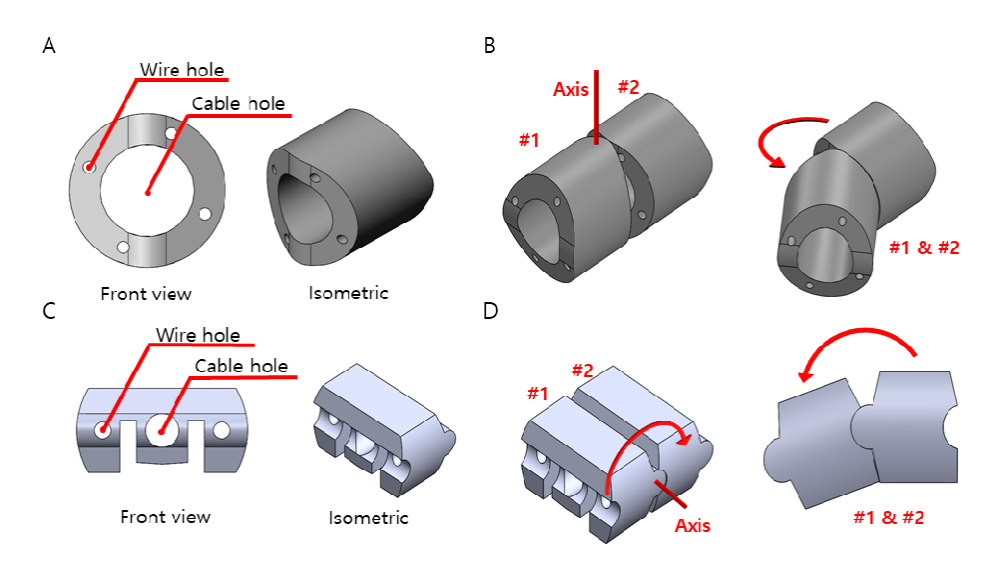
Configuration of motions in the continuum. (A) Structure of the endoscope continuum. (B) Bending motion of the endoscope continuum in action. (C) Structure of the tongue continuum. (D) Bending motion of the tongue continuum in action.

The endoscopic continuum consists of individual continuum segments connected by stranded wires. As shown in Figure 5A, each segment has 4 wire holes for the articulation wire and a big central cable hole for the endoscope camera cable. The tension of the wires is adjusted by winding, and rewinding narrows the lateral gap between segments, therefore generating 2-degree-of-freedom (2DOF) omnidirectional motion [10], as depicted in Figure 5B. Two opposite-sided wire holes in the individual segments are paired to each other, making 4 wire holes in total. Each wire hole pair is responsible for the rotational steering movement between 2 segments. The collectible movements of segments create the steering motion.

**Figure 6:**
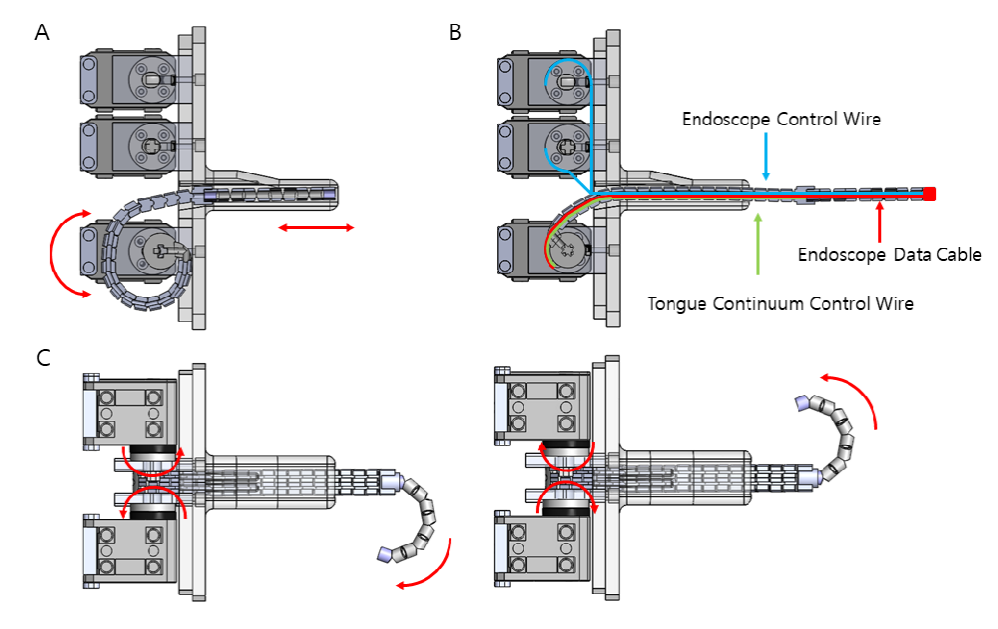
Articulation principle of continuums. (A) Extending and retracting motion resulting from the rolling motion of the tongue continuum winder motor. (B) Arrangement of the inner wire connection of the continuum. (C) Endoscope continuum in motion after extension.

The tongue continuum is quite different. Only having a slope on the front and the back side of the single continuum segment, the tongue continuum has 2 wire holes for a supportive wire on each wing of the individual segment, a central cable hole for the endoscope cable, and 2 slits at the bottom for the articulating wire, as shown in Figure 5C. The tongue continuum can bend up and down (bidirectional), as shown in Figure 5D. In addition, the wire connecting all the tongue continuum parts is not skewed in a specific direction, let alone being connected to a separate motor. Lacking an active driving force of motion, it can only be passively rolled in and out. The tongue continuum flattens the tongue to get the endoscope safely in position. Coordinated with the motor movement, the tongue continuum module can be retracted and extended by rolling it in and out of the mask, as shown in Figure 6.

As shown in Figure 6, the 4 upper motors aligned in rectangular corners are responsible for articulation of the endoscope continuum. The motor positioned at the downmost location in the mask is responsible for the extending and retracting motions of the tongue continuum. The tongue continuum is directly connected to the winder wheel, and the wheel itself serves as the winder for the endoscope module and the tongue continuum’s supporting wire inside the continuum. The wire connected to the tongue continuum is flexible enough to be kept rolled up in the mask when the endoscope is not in use. As the motor turns clockwise, the tongue continuum unit unrolls from the winder, extending forward. While the tongue continuum extends forward, the other 4 motors also correspond to the motion, unrolling the wires to match the length. This results in only the tongue continuum extending forward, without any bending of the endoscope continuum. This motion corresponds with the GO button on the app controller.

**Figure 7:**
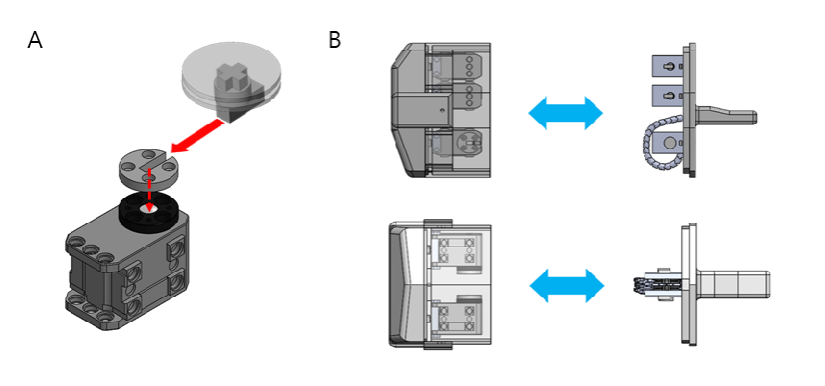
Principle of the key-and-pulley parts, rendered in 3D simulation. (A) Assembly of the key-and-pulley parts. (B) Combining the pulley and motor parts, and key and continuum parts, rendered in a 3D model.

When the tongue continuum is extended to the desired length, the operator can manipulate buttons in the app to control the endoscope continuum. Rolled out with the tongue continuum, the endoscope continuum is connected to separate wires that lead to the winder connected to the 4 upper motors. Each motor is in charge of unidirectional bending of 2DOF motion, that is, a pair of motors corresponds to bidirectional bending motion, as shown in Figure 7.

After imaging, the operator can press the BACK button to turn the motor counterclockwise to make the tongue continuum roll back into the mask. For the pulling motion, while the winder of the bottom motor rotates, like the device articulated in the extending motion, the other 4 motors rotate to wind the wire back up to match the retracting length of the endoscope.

### Pulley and Key

Medical devices in contact with patients must be sterilized and reprocessed by thorough cleaning. However, complex medical devices, especially those with electronic parts, are difficult to sterilize without damaging the electronic parts. The contactless wireless mask endoscope is no exception. Many medical electronic devices overcome this problem by separating the parts in contact with the patient from the noncontact parts using disposable covers like a medical drape. As shown in Figure 7, our device is made of 2 detachable assemblies, a continuum module and a motor assembly module.

For separation, the device needs to connect and convey the force between parts. By employing a key on the pulley, the force from the motors can be transmitted to the continuum module directly. The pulley is designed as a simple disk with a slit that goes from the center of the pulley to the outermost rim, which appears U-shaped, as depicted in Figure 7A. The key is a small rectangular metal rod-like part, which has a small cross on the disk attached to the tip of the long side of the rod. When fully assembled, the key is inserted into the slit of the pulley. Figure 7B shows the key-and-pulley assembly in action. The pulley is attached to the motor wheel by screws (4 holes). The pulley can be easily disassembled from the motor wheel by unscrewing the screws, but when it is screwed on, the pulley and the motor wheel are securely attached. On the other hand, the key is attached directly to the wire winder wheel, which is directly connected to each wire that is responsible for articulation. By sliding the assembled key into the slit of the pulley, the assembled motor unit and the endoscope modules are safely connected.

## Results

### Continuum Contact Force Experiment

The contactless mask endoscope is designed as a laryngoscope, which means the endoscope part has to go over the patient’s tongue, hard palate, and soft palate to finally arrive at the vocal cord. If the flexible continuum is too rigid, it can injure oropharyngeal tissues, resulting in secondary infection. A simple transoral model was created to evaluate the end-effect force when the continuum articulates.

**Figure 8:**
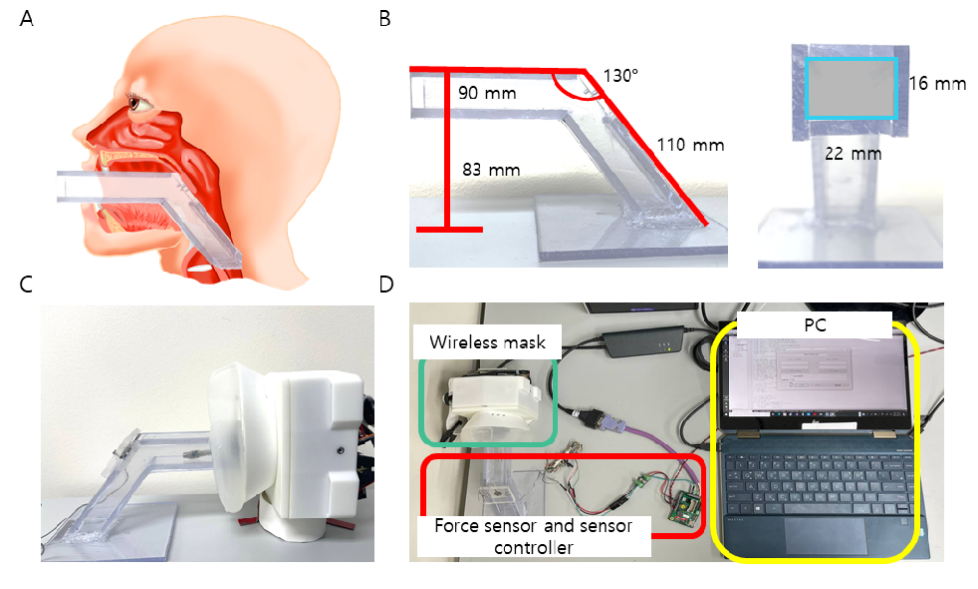
Continuum contact force measurement experiment and structure of the transoral model for the experiment. (A) Comparison of human oropharyngeal anatomy and the transoral model structure. (B) Dimension of the transoral model. (C) Wi-Fi–based contactless wireless mask endoscope positioned with the transoral model for the continuum force measurement experiment. (D) Experimental setting of the continuum tip force measurement.

Assuming the continuum always collides with the oral tract, a force sensor (Honeywell International Inc) was placed where the soft palate is present, since the continuum is most likely to collide with the soft palate. The force against the surface was measured 10 times. The overall experimental setup is presented in Figure 8D. According to studies on oral anatomical structures, the hard palate length is 46.65 to 51.89 mm for adult males and females [11-16], and the soft palate length is 25.3 mm on average. The radius of the esophagus at rest varies from 12.87 to 17 mm. The angle between the soft palate and the esophagus is 130.1° to 137° [17].

Figures 8A and 8B present the oral structures and transoral model constructed from the information about oral structures. The length of the simulated oral part of the transoral model was set to 90 mm, the angle between the oral part and the esophagus was 130°, the cross-sectional area of the simulated esophagus in the transoral model was 16 mm × 22 mm, and the height was set to 83 mm. The contactless wireless mask endoscope was set in the transoral model, as shown in Figure 8C. Without articulating the endoscope continuum, the tip of the endoscope was collided with the force sensor located on the simulated oropharynx wall of the transoral model only using back-and-forth articulation of the tongue continuum. The collision was repeated 11 times with 10-second intervals.

### Wireless Video Transfer Rate Evaluation

**Figure 9:**
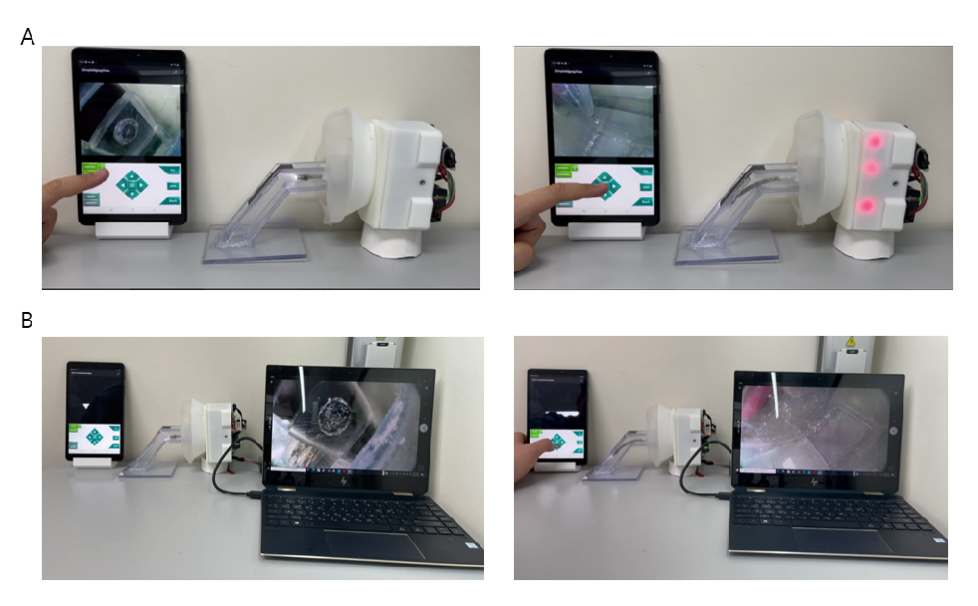
Wireless video transfer rate evaluation experiment. (A) Wireless and (B) wired articulation and video transfer between a contactless wireless mask endoscope and laptop computer. The transferred videos are displayed on the screens of both laptops.

The contactless wireless mask endoscope processes raw video data in the controller and sends them to the Android tablet through an access point. There is a small delay due to the limited hardware specification and the nature of wireless communication. To evaluate the delay in the video broadcast from the endoscope, a comparison experiment was conducted. Figure 9 shows the experimental setup of the wireless and wired endoscope video delay test. From the same camera module, the video was transferred directly from the camera and broadcast from the Raspberry Pi module. Using forward articulating movement as a standard, we compared the frame rate and delays between the 2 videos.

### Wireless Articulation Experiment Using a Phantom

Using a phantom resembling the real-life structure of a patient’s laryngopharyngeal tract, a wireless articulation experiment was conducted to evaluate the practicality of the contactless mask endoscope.

**Figure 10:**
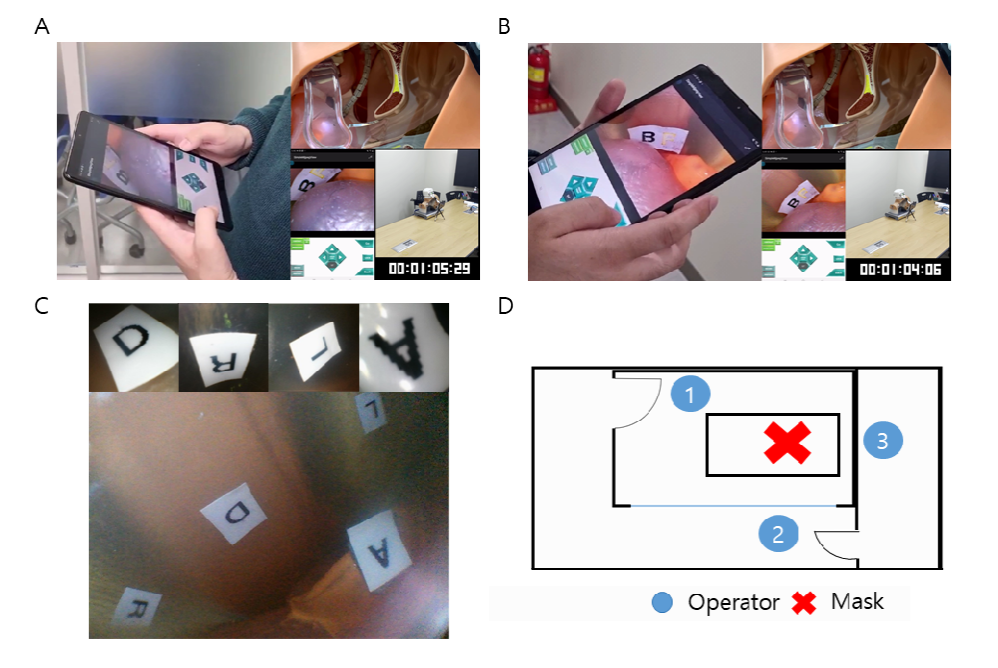
Wireless articulation experiment. Compilation of videos resulting from the experiment conducted outside of the office (A) over a glass partition and (B) behind a concrete wall. Each video is synced to the timestamp on the bottom right of the video. (C) Snapshot taken from each marked position by articulating the endoscope continuum to deliver the tip to the desired position. (D) Position of the mask endoscope and operator for each experiment.

The conjoined experiment involved a contactless mask endoscope, 2 cameras, a phantom, and an operator with an Android tablet. The abstract experimental setup is shown in Figure 10 and Multimedia Appendix 1. The laryngopharyngeal phantom was set at the center of the meeting room. The contactless mask endoscope was applied to the phantom, and the operator was outside the meeting room. The operator began to control the endoscope with the tablet through a wireless connection. The camera simultaneously recorded both sides as the operator controlled the endoscope to take snapshots and videos of stickers attached to the desired parts of the phantom. The process was repeated after changing the operator’s location as follows: in the front of the meeting room, in the corridor, and in the laboratory right next to the meeting room.

**Figure 11:**
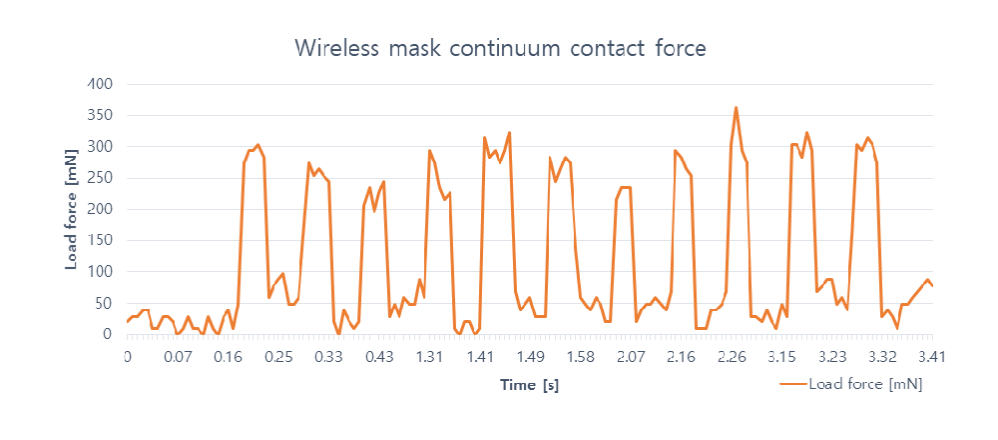
Measured force of the endoscope continuum colliding against the wall of the transoral model.

The results of the continuum contact force experiment are shown in Figure 11. The magnitude of the collision force varied from a minimum of 235 mN (24 gf) to a maximum of 363 mN (37 gf). The average collision force was 296 mN (30.2 gf), which is just 6.5% of that of a commercial endoscope.

**Figure 12:**
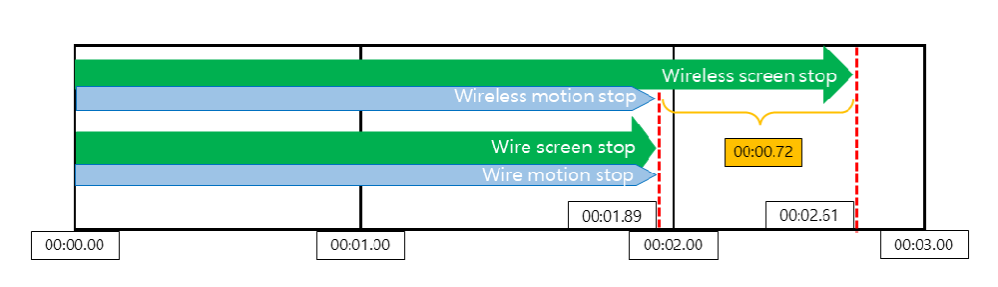
Time lapse of the wireless video transfer rate evaluation experiment.

The results of the wireless video transfer rate evaluation experiment are summarized in Figure 12. Comparing wired connection and wireless communication, wireless video streaming was delayed by 0.72 seconds with the contactless mask endoscope.

In the wireless articulation experiment, we placed marker stickers in the desired areas as shown in Figure 10C and the operator of the mask endoscope controlled the device and examined the video data streamed from it while being spatially separated completely from the patient. The measured time to get the stickers in the video stream was around 1 minute in the desired separated location. From the experiment, we tried to evaluate the articulating range of the endoscope continuum and the capability of the device’s wireless communication that enables users to control the device from a different space. Contactless wireless video streaming was successful within the range of the access point regardless of the presence of any material blocking the way.

In summary, the Wi-Fi–based contactless mask endoscope was controlled and articulated wirelessly, minimizing or completely avoiding possible contact between patients and device operators. By minimizing contact, contactless medical devices can protect health care workers from infectious diseases like COVID-19.

## Discussion

### Principal Findings

In the current COVID-19 pandemic era, physicians run risks when they evaluate a patient with fever of unknown origin or with laryngeal symptoms, such as dyspnea, hoarseness, and dysphagia. However, these symptoms are associated with laryngeal obstruction and could be exacerbated when adequate evaluation and treatment are lacking. The proposed wireless device would help protect physicians from possible contagious diseases while allowing them to promptly evaluate patients.

Previously, our team developed a mobile-based USB on-the-go (OTG) endoscope that can be articulated using an Android mobile app [10]. The Wi-Fi–based contactless mask endoscope has several advantages over the USB OTG endoscope. First, it can be controlled over a wireless network, enabling the operator to position himself/herself freely, unlike the USB OTG endoscope, for which the operator has to be close to the patient. Second, the operator of the Wi-Fi–based mask endoscope can attach the endoscope via a strap, unlike the USB OTG endoscope, which has to be held with the cell phone. This has the secondary benefit of decreasing the load on the operator’s wrist and hand, providing a better user experience. Finally, modulization of the Wi-Fi–based contactless mask endoscope makes it easier to replace parts, simplifying maintenance.

As individual parts can be replaced rather than replacing the whole module, maintenance costs are reduced. The price of the USB OTG endoscope was US $473, which was one of the main advantages of the device, as it was intended to be used in underdeveloped countries. Table 1 shows the price of the Wi-Fi–based contactless mask endoscope. Considering that the cost of the 3D-printed modules can be reduced with mass production, in addition to the reduced maintenance costs, the price may be better than that of the USB OTG endoscope [10].

**Table 1:** Prices of hardware parts of the Wi-Fi–based contactless wireless mask endoscope.

Hardware	Price (US $)
XL320 * 5ea	97.8
3.9-mm camera module	170
3D print of the case	68.96
3D print of the tongue and endoscope continuum	152.88
Controller	60.02
Wires, etc	49.27
Total	598.93

A snake-like continuum is commonly used in flexible endoscope articulation, enabling multiple degrees of motion for clinical needs. However, complex articulation was historically reserved for larger endoscopes. Endoscopes with multiple kinds of articulating units for flexible parts have some advantages. First, they can perform different functions, such as adjusting the mechanical force applied to the articulating units to ensure patient safety [18]. Second, the multiple kinds of articulating units can exert different kinds of motion for each kind of unit, enabling users to control dynamic motions [19]. While the endoscope continuum of this contactless wireless mask endoscope serves as the conventional endoscope articulating unit, the tongue continuum has 2 other functions. First, its broad shape and stiff mechanical property flattens the tongue. During endoscopy, especially of the stomach and upper respiratory organs, the tongue gets in the way of the endoscope, hindering its movement. Therefore, it is recommended that the examiner use anesthesia before endoscopy to keep the tongue out of the way. A mouthpiece with a tongue presser can be used [20]. The tongue continuum of the contactless wireless mask endoscope also serves as a tongue presser, making it easier to enter the desired area. Second, the tongue continuum itself works as a support for the regular continuum. If the snake-like continuum is too long, due to capstan friction, forces on the end point cannot drive the end point as intended [18]. Adding a tongue continuum shortens the length of the snake-like continuum, making it easier to relay force to the tip of the endoscope and enabling precise control. In conclusion, having a semirigid tongue continuum has the advantage of articulation control and safe delivery of the endoscope tip to the desired area.

The system’s modularity also reduces contamination concerns. Endoscopes are more prone to cross-infection than other noninvasive examination devices because endoscopy involves mucosal contact. The endoscope module cannot be sterilized by autoclaving because the electronics within it are sensitive to heat, pressure, and moisture. Given that autoclaving is one of the easiest and most reliable methods of disinfection, the endoscope module must be changed regularly regardless of the cost.

To solve the contamination problem, 2 major innovations were incorporated. First, a detachable endoscope module was used. With the key-and-pulley part inside the mask, the endoscope module is fixed to the motor. When the operator decides to separate the mask and the endoscope, the 2 parts can be separated by disassembling the key-and-pulley part. The inner space of the mask can be easily accessed by opening the outer lid. Separation of parts also creates a gap between the parts, where an anticontaminant cover sheet can be applied. Therefore, by replacing the wheel, the possibly contaminated continuum and endoscope modules can be separated from the mask module, and the mask can be kept clean. The second innovation is that of a simplified inner structure. For a larger endoscope, the sheath has to have some kind of contact point because of the channel in the endoscope. However, the contactless wireless mask endoscopic system does not have such a channel, and therefore, the sheath can be simple, like a condom. Without a channel, it is much easier to sterilize the smooth surface, and there is much less chance of contamination because of the small contact point.

For conventional endoscopes, portable devices with a wireless connection are not preferred for multiple reasons. First, a wireless connection has a limited data transfer rate. The Bluetooth 5.0 connection has a 1 Mbps data transfer rate for low-energy Bluetooth [21]. The 1 Mbps rate of Bluetooth 5.0 can barely cover a standard-frame-rate 360p video stream [22]. A Wi-Fi connection is more generous in terms of the bit rate, and 802.11n families can go up to 200 Mbps, enough to stream 4K videos [22,23]. Second, since endoscopy is a medical procedure for which many trained health care experts are needed, there is less need to separate the endoscope and the operating system. However, due to the COVID-19 pandemic, there is a possibility of infecting entire medical facilities during endoscopy. Therefore, the need for separation of the operator and patient has emerged.

The current focus mainly revolves around contactless endoscopy that is performed in close proximity but remains in the clinic. While the practitioner is in a separate room, a patient can follow instructions to wear the mask. When the mask is properly worn, endoscopy can be performed remotely using the smartphone app. Should an emergency occur, practitioners waiting outside the room can interrupt the procedure and ensure the patient’s safety. Otherwise, the practitioner can utilize the internet to extend the range of communication. The endoscope streams the video from the patient to the practitioner. Theoretically, by setting up a public IP address and encrypted data transfer, users can safely control the endoscope over the internet from many kilometers away. Subsequent research will involve safety and clinical trials performed at Asan Medical Center using an improved version of the articulating endoscope system.

The designed laryngoscope goes over soft areas that can trigger a gag reflex. The oropharyngeal area is sensitive, so it can detect down to 2.07 mmHg of pressure [24,25]. A gag reflex can be triggered by a slight touch of the triggering area [25]. Generally, laryngoscopy involves carefully avoiding the areas that would trigger a gag reflex [26] or using local anesthesia [27-29]. In particular, noninvasive local anesthesia can be applied easily with fewer side effects and fewer tissue injuries. Therefore, in practice, the device can be accompanied with local anesthetic application by hand or machine. Lidocaine nebulizer, spray, or ingestion would help ameliorate the gag reflex.

### Limitations

While making the Wi-Fi–based contactless mask endoscope, we faced a few problems mainly due to hardware limitations. The 3D-printed parts did not achieve a tight seal and allowed air to flow through the motor box. For clinical application, improved seals combined with a filtered ventilation port would reduce the risk of aerosol transmission. Further hardware limitations mandated a tradeoff between device size and capabilities. Because the mask needs to be worn like a conventional respiratory mask, the dimensions of the attached controller and motor parts were minimized to enhance the patient’s experience (Table 2). The lack of space and need to downsize the controller also lowered the performance of the control system, resulting in delays and reduced video-processing performance, which led to a 0.72-second delay in wireless video streaming.

For future research, we will compensate the space limitation in hardware with better performance, while maintaining the portable point of care–like nature of the device. In particular, the device may be widened to incorporate an additional channel for biopsy or to increase patient comfort.

**Table 2:** Dimensions of the Wi-Fi–based contactless mask endoscope.

Part and dimension		Value (mm)
**Mask**		
	Width (with cushion part)	99
	Height (with cushion part)	120
	Depth (with cushion part)	47
	Width (without cushion part)	90
	Height (without cushion part)	118
	Depth (without cushion part)	26
**Controller and motor box**		
	Width	97
	Height	114
	Depth	77
**Hardware**		
	Width	99
	Height	122
	Depth	123

### Conclusions

We developed a contactless wireless mask endoscopy and diagnosis system to be used during the COVID-19 pandemic. Using Wi-Fi and an Android mobile device, we successfully articulated the endoscope and took snapshots and videos of the patient’s vocal cord. The device can separate health care workers from patients, while enabling diagnosis of internal parts of the body that cannot be reached without endoscopy. In conclusion, the contactless wireless mask endoscope can help minimize chances of contamination, further minimizing the impact of the pandemic in the medical field.

## Appendix

Multimedia Appendix 1 Contactless wireless endoscope mask in action. Left: operation of the endoscope from an adjacent hallway. Right top: close-up view of endoscope articulation in the laryngopharyngeal phantom. Right bottom left: screen capture of the mobile app in action. Right bottom right: view of the endoscope mask installed on the phantom.

## Abbreviations

2DOF: 2-degree-of-freedom
OTG: on-the-go

## References

[1] Nguyen L, Drew D, Graham MS, Joshi AD, Guo C, Ma W, Mehta RS, Warner ET, Sikavi DR, Lo C, Kwon S, Song M, Mucci LA, Stampfer MJ, Willett WC, Eliassen AH, Hart JE, Chavarro JE, Rich-Edwards JW, Davies R, Capdevila J, Lee KA, Lochlainn MN, Varsavsky T, Sudre CH, Cardoso MJ, Wolf J, Spector TD, Ourselin S, Steves CJ, Chan AT, Albert CM, Andreotti G, Bala B, Balasubramanian BA, Beane-Freeman LE, Brownstein JS, Bruinsma FJ, Coresh J, Costa R, Cowan AN, Deka A, Deming-Halverson SL, Elena Martinez M, Ernst ME, Figueiredo JC, Fortuna P, Franks PW, Freeman LB, Gardner CD, Ghobrial IM, Haiman CA, Hall JE, Kang JH, Kirpach B, Koenen KC, Kubzansky LD, Lacey, Jr JV, Le Marchand L, Lin X, Lutsey P, Marinac CR, Martinez ME, Milne RL, Murray AM, Nash D, Palmer JR, Patel AV, Pierce E, Robertson MM, Rosenberg L, Sandler DP, Schurman SH, Sewalk K, Sharma SV, Sidey-Gibbons CJ, Slevin L, Smoller JW, Steves CJ, Tiirikainen MI, Weiss ST, Wilkens LR, Zhang F (2020). Risk of COVID-19 among front-line health-care workers and the general community: a prospective cohort study. The Lancet Public Health.

[2] Kim SI, Lee JY (2020). Walk-Through Screening Center for COVID-19: an Accessible and Efficient Screening System in a Pandemic Situation. J Korean Med Sci.

[3] Lavine JS, Bjornstad ON, Antia R (2021). Immunological characteristics govern the transition of COVID-19 to endemicity. Science.

[4] Frangenheim H (1988). History of Endoscopy. Gynaecological Endoscopy.

[5] Kaunitz JD (2014). The fruits of fiber: the invention of the flexible fiberoptic gastroscope. Dig Dis Sci.

[6] Kovaleva J, Peters FTM, van der Mei HC, Degener JE (2013). Transmission of infection by flexible gastrointestinal endoscopy and bronchoscopy. Clin Microbiol Rev.

[7] Soetikno R, Teoh AY, Kaltenbach T, Lau JY, Asokkumar R, Cabral-Prodigalidad P, Shergill A (2020). Considerations in performing endoscopy during the COVID-19 pandemic. Gastrointest Endosc.

[8] Minelli Grazioli L, Milluzzo SM, Spada C (2020). Safe endoscopy during the COVID-19 pandemic. Gastrointest Endosc.

[9] Marlicz W, Ren X, Robertson A, Skonieczna-Żydecka K, Łoniewski I, Dario P, Wang S, Plevris JN, Koulaouzidis A, Ciuti G (2020). Frontiers of Robotic Gastroscopy: A Comprehensive Review of Robotic Gastroscopes and Technologies. Cancers (Basel).

[10] Moon Y, Oh J, Hyun J, Kim Y, Choi J, Namgoong J, Kim J (2020). Cost-Effective Smartphone-Based Articulable Endoscope Systems for Developing Countries: Instrument Validation Study. JMIR Mhealth Uhealth.

[11] Mustafa AG, Tashtoush AA, Alshboul OA, Allouh MZ, Altarifi AA (2019). Morphometric Study of the Hard Palate and Its Relevance to Dental and Forensic Sciences. Int J Dent.

[12] Mahendran S, Thenmozhi MS (2017). Sexual Dimorphism of Adult Human Palate by its Dimensions in South Indian Dry Skulls. International Journal of Pharmaceutical Sciences Review and Research.

[13] Jotania B, Patel SV, Patel S, Patel PM, Patel SA, Patel K (2013). Morphometric analysis of hard palate. International Journal of Research in Medicine.

[14] Fauzi NQBA, Eapen B (2017). Patient management education for dentists. International Journal of Current Research.

[15] Fitch WT, Giedd J (1999). Morphology and development of the human vocal tract: a study using magnetic resonance imaging. J Acoust Soc Am.

[16] Badshah M, Soames R, Khan MJ, Hasnain J (2018). Morphology of the human hard palate: a study on dry skulls. Italian Journal of Anatomy and Embryology.

[17] Joseph AA, Elbaum J, Cisneros GJ, Eisig SB (1998). A cephalometric comparative study of the soft tissue airway dimensions in persons with hyperdivergent and normodivergent facial patterns. J Oral Maxillofac Surg.

[18] Chen Y, Tanaka S, Hunter IW (2010). Disposable endoscope tip actuation design and robotic platform.

[19] Yoon HS, Yi BJ (2009). A 4-DOF flexible continuum robot using a spring backbone.

[20] Lee S, Park Y, Cho S, Kang J, Lee D (2015). Technical skills and training of upper gastrointestinal endoscopy for new beginners. World J Gastroenterol.

[21] Daudov IM, Orobey MN, Ignatev IV (2021). Bluetooth based technology for industrial personnel local positioning. IOP Conf. Ser.: Mater. Sci. Eng.

[22] Recommended upload encoding settings. Google Support.

[23] Chan H (2007). Comparing wireless data network standards.

[24] Bearelly S, Cheung SW (2017). Sensory Topography of Oral Structures. JAMA Otolaryngol Head Neck Surg.

[25] Aviv JE, Martin JH, Jones ME, Wee TA, Diamond B, Keen MS, Blitzer A (1994). Age-related changes in pharyngeal and supraglottic sensation. Ann Otol Rhinol Laryngol.

[26] Beales PH, Al-Khaled MJ (1980). Procedures in practice. Laryngoscopy. Br Med J.

[27] Kamran M, Qamar R (2016). An easy and effective way to reduce gag during orthodontic impression recording. Pakistan Orthodontic Journal.

[28] Means CR, Flenniken IE (1970). Gagging--a problem in prosthetic dentistry. J Prosthet Dent.

[29] Singh S, Ali FM, Nazirkar G, Dole VK, Gaikwad B (2013). Gag- Etiology and Its Skillfull Management- A Review. Journal of Evolution of Medical and Dental Sciences.

